# Risk factors associated with local complications of erysipelas: a retrospective study of 152 cases

**DOI:** 10.11604/pamj.2017.26.66.11096

**Published:** 2017-02-05

**Authors:** Hicham Titou, Christelle Ebongo, Elarbi Bouati, Mohammed Boui

**Affiliations:** 1 Dermatology Department, Hospital Military Instruction of Mohamed V, Hay Riad, 10000, Rabat, Morocco University Mohammed V, Rabat, Morocco; 2 Dermatology Department, Hospital Ibn Sina, Rabat, Morocco; 3 Department of Social Medicine, Public Health and Legal Medicine, Faculty of Medicine and Pharmacy, University Mohammed V, Rabat, Morocco

**Keywords:** Erysipelas, complications, risk factors, infection, dermohypodermitis

## Abstract

Erysipelas is a common skin infection. Hemorrhagic, bullous, abcessing and necrotic lesions are the major local complications. However, their occurrence factors are not clearly known. The aim of this study is to identify the risk factors associated with the occurrence of local complications of Erysipelas. Medical records from all patients hospitalized with local complications of erysipelas admitted to the Military Hospital of Rabat between 2005 and 2015, were retrospectively studied. Using an univariate and multivariate statistical study, the main characteristics were compared with those from patients with erysipelas without local complications. In total, 152 patients were analysed, of whom 72 had local disease complications. Using univariate analysis, the factors significantly associated with disease complications were found to be: age ≤ 50 years, female gender, heart disease, smoking, taking antibiotics or non-steroid anti-inflammatory drug before hospitalization, and accelerated sedimentation rate. However, in multivariate analysis, taking antibiotics before hospitalization (OR 5.15, 95% CI 1.28 to 20.72, P = 0.01) and accelerated sedimentation rate (OR 5, 15, 95% CI 1.00 to 1.06, P = 0.001) were the only independent factors associated with complicated erysipelas. Our study showed that prior antibiotics taking and higher sedimentation rate are independent risk factors for local complications of erysipelas. Patients with these characteristics should be carefully evaluated and monitored.

## Introduction

Erysipelas is an infectious disease of the dermis and subcutaneous tissue commonly caused by streptococci [1]. It is a clinical form of acute cellulitis [2]. Clinically it is characterized by the acute onset of local signs of inflammation such as erythema, oedema, pain and heat. In its classic form it is accompanied by systemic signs such as fever, chills and malaise and sometimes nausea and vomiting [3, 4]. An increase of inflammation biological parameters is frequent such as leukocytosis with predominance of neutrophils, and increased C-reactive protein (CRP) [5]. Erysipelas can be serious but rarely fatal. Erysipelas has a rapid and favorable response to antibacterial therapy [6, 7]. Systemic complications are very rare: sepsis was reported in 2-5% of patients in large series [7, 8]. Local complications are more frequent occurring in one third of patients hospitalized for erysipelas [9] and they are mainly abscess formation, necrosis, bubbles and hemorrhagic purpura. These complications are associated with a more severe condition [10, 11]. In this study, we reviewed all cases of erysipelas recorded in our hospital within a period of 10 years with the aim of assessing possible risk factors for patients developing local complications.

## Methods

### Study protocol

The study was a retrospective analysis of clinical records in all hospitalized patients with erysipelas in the Department of Dermatology of the Military Hospital of Rabat, from 2005 to 2015. Erysipelas was defined as a skin infection of sudden onset with a red indurated expanding plaque with a distinct border isolated or associated with one or more of the following: the identification of an infection door, a sensitive satellite lymphadenopathy or fever. All patients with local complications of erysipelason admission or during hospitalization including haemorrhagic, bullous, abscessing or necrotic lesions, who were admitted to the hospital during the same period, were included in the study.

Patient information was recorded, including gender, age, temperature, lesion site, lymph-node involvement, fever duration, days of hospitalization, number of relapses before complications, the initial response to antibiotic therapy and presence of coexisting diseases. Local factors such as presence of ulcers, local surgery, tineapedis, varicose veins, injury and lymphoedema were also recorded as were laboratory parameters, including white blood cell (WBC) count, erythrocyte sedimentation rate and C-reactive protein levels on admission and at discharge. In most cases, no bacteriological evidence was requested.

All cases were divided into two groups: those with uncomplicated and those with complicated erysipelas. The group of complicated erysipelas included all patients with bullous, abscessing, hemorrhagic or necrotizing forms. We compared the clinical and laboratory characteristics between the two groups.

### Statistical analysis

The collected data were entered and analyzed by SPSS 18.0 for Windows software. Risk factors for complicated erysipelas were assessed using univariate analysis based on the Mann-Whitney test or the Student t-test for continuous and the Chi 2 test or Fisher exact test for qualitative variables. The results were considered statistically significant for a p-value ≤ 0.05. Variables with crude OR ≤ 0.20 were selected for multivariate analysis to identify potential independent risk factors.

## Results

In total, 152 patients were included in the analysis, of whom 72 (47%) had a local complication. The middle age was 54 ± 17 years with a sex ratio M/F= 3/1. 45 patients (29%) had a past medical history of erysipelas among which 20 patients (13%) with more than two recurrences. The most frequent local complications were bullae (42 patients: 27%), followed by haemorrhagic lesions (31: 20%), abscesses (17: 11%) and necrosis (9: 5%). 23 patients (15 %) had a combination of complications. In our study, 48 % of hospitalized patients were apyretic before treatment.

The presence of entry portals was frequently found such as tinea pedis (78%), local surgery (13%), eczema (1%), and infected ulcer (1%). Most of our patients were treated without bacteriological confirmation. In the univariate analysis of the general factors, age ≤ 50 years (48% vs. 66%, P = 0.02), female gender (10% vs. 18%, P = 0,01) and smoking (46% vs. 29%, p = 0.03) were found to be positively associated with complicated erysipelas (Table 1). Among the various comorbidities, only heart disease was a factor predisposing to complications of erysipelas (27% vs. 47%, P = 0.01). Empirical antibiotic therapy before hospitalization (15% vs. 31%, P = 0.01), and anti-inflammatory drugs taking (17% vs. 31%, p = 0.05) appeared as factors associated with occurrence of local complications of erysipelas. An accelerated erythrocyte sedimentation rate under a threshold of 50 mm / h (12% vs. 25%, p = 0.02) was positively associated with the risk of local complications. As a result, days of hospitalization were increased in patients with complicated erysipelas (p <0.05).

**Table 1:** Univariate analysis of risk factors for local complications of erysipelas

Caracteristics	Non complicated n=80	Complicated n=72	P
Ageinyears (mean±SD)	51±17	57±16	0,02^+^
Age≥50	39(48)	48(66)	0,02^+^
M/F	67/13	47/25	0,01^+^
Site			
FaceUpper	7(8)	0	
Limbs Lower	4(5)	1(1)	
limbs	69(86)	71(98)	
Pasthistory			
Diabetes	26(32)	27(37)	0,51
Cardiopathy	22(27)	34(47)	0,01^+^
Chronic renal	3(3)	4(5)	0,70
failure	44(55)	49(68)	0,11
Obesity	6(7)	3(4)	0,50
Neoplasia	37(46)	21(29)	0,03+
Smoking	11(13)	8(11)	0,64

M, male; F, female; Data are n (%) unless otherwise indicated.

+ Significant.

In our study, obesity was most frequently found in the group with complicated erysipelas (55% vs. 68%, P = 0.11), although this difference was not statistically significant. On admission leukocytosis (WBC> 10,000 / microl) was observed in 32% of cases, and accelerated sedimentation rate (ESR> 50mm / h) in 18% of cases. The percentage of patients with local complications responding to an initial empirical antibiotic treatment given was slightly lower than patients without complications (96% vs. 95%, P = 0.89) (Table 2).

**Table 2:** Univariate analysis of clinical and biological characteristics of base

	Non complicated n=80	Complicated n=72	P
Presence of fever	38(47)	41(56)	0,24
Lymphadenopathy	32(40)	24(33)	0,39
Biological parameters (mean±SD)			
WBC,µl	12510±8600	10383±9050	0,39
WBC>10000,µl	25(31)	25(34)	0,84
N,µl	6999±6100	7259±6000	0,70
ESR,mm/h	38±28,9	43±24,1	<0,05^+^
VS≥50mm/h	10(12)	18(25)	0,02^+^
CRPmg⁄dl	64±42	96±53	0,20
Days of hospitalization (mean±SD)	8,2±4,7	10,7±5,7	<0,05^+^
Good response to initial treatment	77(96)	69(95)	0,89
Recurrences	26(32)	19(26)	0,41

WBC, white blood cell count ; N, neutrophiles ; ESR, erythrocyte sedimentation rate; CRP, C-reactive protein; Results are in number n (%)

+ statistical significative

Finally, in the multivariate analysis (Table 3) empirical antibiotherapy before hospitalization (OR = 5.15, 95% CI 1.28 - 20.72, P = 0.01) and accelerated sedimentation rate (OR = 5.15, 95% CI 1.00 -1.06, p <0.05) appeared to be factors independently associated with complicated erysipelas.

**Table 3:** Multivariate analysis of risk factors of local complications of erysipelas

	Odds ratio	95% CI
Antibiotherapy before hospitalization	5,15	1,28 – 20,72
ESR on admission	1,03	1,00 – 1,06

ESR, erythrocyte sedimentation rate; CI, confidence interval

## Discussion

In this study, we investigated patients with erysipelas for risk factors predisposing to the development of local complications. One of the main findings was that prior empirical antibiotics taking and increased erythrocyte sedimentation rate at admission are the only independent risk factors. This suggests firstly that poor initial management plays an important role in the occurrence of complications, and secondly the erythrocyte sedimentation rate can be a good biomarker ofcomplication risk and hospital criterion for rigorous monitoring and specific care.

Complications occurred in 47% of patients in our series, which is consistent with other series 31% [9], 52% [12]. This rate may reflect an overestimation of complications in patients with erysipelas because it only deals with hospitalized patients. Those complications result in an increased length of stay (8.2 vs.10, 7 P <0.05). Reflecting the discomfort of patients and the socio economic impact. In our practice, there was an extension of the duration of antibiotic therapy in complicated patients, which was demonstrated in previous studies [9, 12]. Schrock JW et al. concluded in their study that patients with complicated erysipelas require antibiotic treatment intensification [13]. We did not find significant differences in obesitybetween the 2 groups in our series, in contrast to a previous study of Krasagakis et al. [9]. Obesity is a known risk factor for erysipelas [14, 15]. It is considered as a severity marker indicating hospitalization [10].

Our study revealed statistically significant difference between the two groups regarding age > 50, female gender, smoking and cardiovascular history. These results are similar to those reported in some series in the litterature [9, 12, 16, 17]. These factors have disappeared in the multivariate analysis. In fact, different underlying diseases and risk factors are described as present in a population with complicated erysipelas but only some of them have a proven role in the pathogenesis of these local complications. The ability to detect other risk factors was limited by the small number of cases of complicated erysipelas (n = 72), despite the large sample size (n = 152). The prior empirical antibiotherapy was an independent factor for complicated erysipelas in our series. It was mostly neither an inappropriate antibiotic nor insufficient doses.

Nonsteroidal anti-inflammatory drugs taking was significantly associated with complicated forms in univariate analysis before disappearing in multivariate analysis. This inadequate care can be explained partly by the initial absence of fever that could move the attention of the clinician to another local skin disease. In common practice, there is the persistence of local signs of inflammation after several days of treatment, which may be related to inflammatory processes without persistence of microorganisms in the infected site. A better understanding of the natural history of the disease should help determine the best duration of antibiotic treatment and whether or not an anti-inflammatory treatment.

The standardization delay in laboratory parameters (ESR and WBC) for complicated forms has been shown in previous studies [9, 17]. Our study is retrospective, which limited us to analyze this factor. Although we found that an increased erythrocyte sedimentation rate at the admission is an independent risk factor for local complications. Lazzarini et al. suggest in their study that the sedimentation rate at admission could be a potential indirect marker of disease severity [17].

Some authors have proposed criteria for hospitalization for patients with erysipelas [16, 18]. Data from our study may contribute to these efforts in order to adapt the therapeutic approach and reduce the frequency of complications.

## Conclusion

Erysipelas is a common skin infection which diagnosis is clinical. Local complications are common and increase its socio-economic cost. Identifying patients at risk for these complications remains a challenge for clinicians. Our study suggests that the rate of sedimentation and empirical antibiotic therapy prior to admission are independent risk factors for complicated erysipelas. Patients with these early indicators should be hospitalized for close monitoring and proper care.

### What is known about this topic

- The risk factors predisposing patients for local complications are not fully known;
- Local complications of erysipelas can pose diagnostic and therapeutic challenges, increasing morbidity and healthcare costs.

### What this study adds

- The present study reveals the rate of sedimentation and empirical antibiotic therapy prior to admission as an independent risk factor for local complications of erysipelas;
- Patients with these early indicators should be hospitalized for close monitoring and proper care.

## References

[1] Grosshans EM (1993). The red face: Erysipelas. Clin Dermatol..

[2] Bernard P, Bedane C, Mounier M, Denis F, Bonnetblanc JM (1995). Dermohypodermites bacteriennes de I'adulte: incidence et place de l'étiologie streptococcique. Ann DermatolVenereol..

[3] Bisno AL, Stevens DL (1996). Streptococcal infections of skin and soft tissues. N Engl J Med..

[4] Bonnetblanc JM, Bedane C (2003). Erysipelas: recognition and management. Am J Clin Dermatol..

[5] Linke M, Booken N (2015). Risk factors associated with a reduced response in the treatment of erysipelas. J Dtsch Dermatol Ges..

[6] Crickx B (2001). Erysipèle: évolution médicale sous traitement: Complications. Ann Dermatol Venereol..

[7] Jorup-Rönström C (1986). Epidemiological, bacteriological and complicating features of erysipelas. Scand J Infect Dis..

[8] Chartier C, Grosshans E (1990). Erysipelas. Int J Dermatol..

[9] Krasagakis K, Samonis G, Valachis A, Maniatakis P, Evangelou G, Tosca A (2011). Local complications of erysipelas: a study of associated risk factors. Clin Exp Dermatol..

[10] Picard D, Klein A, Grigioni S, Joly P (2013). Risk factors for abscess formation in patients with superficial cellulitis (erysipelas) of the leg. Br J Dermatol..

[11] Schrock JW, Laskey S, Cydulka RK (2008). Predicting observation unit treatment failures in patients with skin and soft tissue infections. Int J Emerg Med..

[12] Bartholomeeusen S, Vandenbroucke J, Truyers C, Buntix F (2007). Epidemiology and comorbidity of erysipelas in primary care. Dermatology..

[13] Dupuy A, Benchikhi H, Roujeau JC, Bernard P, Vaillant L, Chosidow O (1999). Risk factors for erysipelas of the leg (cellulitis): case-control study. BMJ..

[14] Musette P, Benichou J, Noblesse I, Hellot MF, Carvalho P, Younget P (2004). Determinants of severity for superficial cellulitis (erysipelas) of the leg: a retrospective study. Eur J Intern Med..

[15] Concheiro J, Loureiro M, González-Vilas D, García-Gavín J, Sánchez-Aguilar D, Toribio J (2009). Erysipelas and cellulitis: a retrospective study of 122 cases. Actas Dermosifiliogr..

[16] Lazzarini L, Conti E, Tositti G, deLalla F (2005). Erysipelas and cellulitis: clinical and microbiological spectrum in an Italian tertiary care hospital. J Infect..

[17] Dupuy A (2001). Epidémiologie descriptive et connaissance des facteurs de risque de l’érysipèle. Ann Dermatol Venereol..

[18] Carratalà J, Rosón B, Fernández-Sabé N, Shaw E, del Río O, Rivera A (2003). Factors associated with complications and mortality in adult patients hospitalized for infectious cellulitis. Eur J Clin Microbiol Infect Dis..

